# Long Non-coding RNAs in Tuberculosis: From Immunity to Biomarkers

**DOI:** 10.3389/fmicb.2022.883513

**Published:** 2022-05-11

**Authors:** Xianyi Zhang, Chan Chen, Yuzhong Xu

**Affiliations:** ^1^The Second School of Clinical Medicine, Southern Medical University, Guangzhou, China; ^2^The People’s Hospital of Baoan Shenzhen, Southern Medical University, Shenzhen, China

**Keywords:** tuberculosis, lncRNA, immune response, diagnosis, biomarker

## Abstract

Tuberculosis (TB) caused by Mycobacterium tuberculosis (Mtb) is the leading lethal infectious disease with 1.3 million deaths in 2020. Despite significant advances have been made in detection techniques and therapeutic approaches for tuberculosis, no suitable diagnostic tools are available for early and precise screening. Many studies have reported that Long non-coding RNAs (lncRNAs) play a regulatory role in gene expression in the host immune response against Mtb. Dysregulation of lncRNAs expression patterns associated with immunoregulatory pathways arose in mycobacterial infection. Meanwhile, host-induced lncRNAs regulate antibacterial processes such as apoptosis and autophagy to limit bacterial proliferation. In this review, we try to summarize the latest reports on how dysregulated expressed lncRNAs influence host immune response in tuberculosis infection. We also discuss their potential clinical prospects for tuberculosis diagnosis and development as molecular biomarkers.

## Introduction

LncRNAs, frequently defined as non-protein-coding transcripts greater than 200 nucleotides in length (McFadden and Hargrove, 2016), were initially thought to be “transcriptional noise” (Ulitsky and Bartel, 2013). Recent studies have unraveled the biogenesis of lncRNAs, which are distinct from that of mRNAs. Those lncRNAs, generally display lower sequence conservation (Pang et al., 2006) and contain fewer exons (Washietl et al., 2014; Guo et al., 2020), and their functions are typically linked to their specific subcellular localizations (Statello et al., 2021). In addition, lncRNAs have been reported to include small open reading frames (SMORFs) encoding short functional peptides (Kim et al., 2014; Huang et al., 2017; Fathizadeh et al., 2020; Zhu et al., 2020). Accumulated evidence demonstrates that they are emerging as essential regulators in gene regulation, such as chromatin modification, mRNA stability, transcriptional, translation and post-translation activation/inhibition (Quinn and Chang, 2016; Yao et al., 2019). Meanwhile, lncRNAs have various regulatory functions in biological processes, including apoptosis (Heydarnezhad Asl et al., 2022).

Tuberculosis, one of the leading lethal infectious diseases worldwide caused by Mtb, has been considered a public health emergency that concerns the world. According to the WHO global TB report, there were 5.8 million newly sick and 1.5 million TB-related deaths in 2021 (WHO, 2021, no date). Approximately one-third of the world’s population has a latent TB infection (LTBI), and about 10% can develop active tuberculosis (ATB) with impairment in the immune system (Pai et al., 2016). In the host-pathogen interactions, intracellular survival and replication of Mtb after macrophage phagocytosis leads to an immune response that converges on granuloma formation and/or disease (McCaffrey et al., 2022; Medley et al., 2022). The host cells have adopted a series of clearance mechanisms to facilitate intracellular bacterial killing, such as apoptosis, autophagy, inflammation, and macrophage polarization. Mtb has also evolved with a set of almost perfect immune escape mechanisms to evade the host immune system (Stanley and Cox, 2013). Growing evidence has delineated that the expression of many lncRNAs is involved in TB with a definitive role in orchestrating the biological processes from immune response to host-pathogen interactions. In addition, tissue-specific and condition-specific expression patterns suggest that lncRNAs provide potential biomarkers (Statello et al., 2021). Therefore, in-depth elucidation of the effect and mechanism of lncRNAs may develop clinical prospects for precise TB diagnosis and treatments (Lyu et al., 2021; Wei et al., 2021). In this paper, we review the progress of lncRNA roles in Mtb infection from host immunity to biomarkers and discuss its potential in clinical diagnosis.

## Regulatory Mechanisms of Long Non-Coding Rnas

Depending on specific interactions with DNA, RNA, and proteins, lncRNAs can modulate diverse biological processes through complex and diverse mechanisms, such as execute-as signals, decoys, guides, scaffolds to regulate target genes (Wang and Chang, 2011; Kazemzadeh et al., 2015; Statello et al., 2021).

### DNA Level Regulation

**FIGURE 1:**
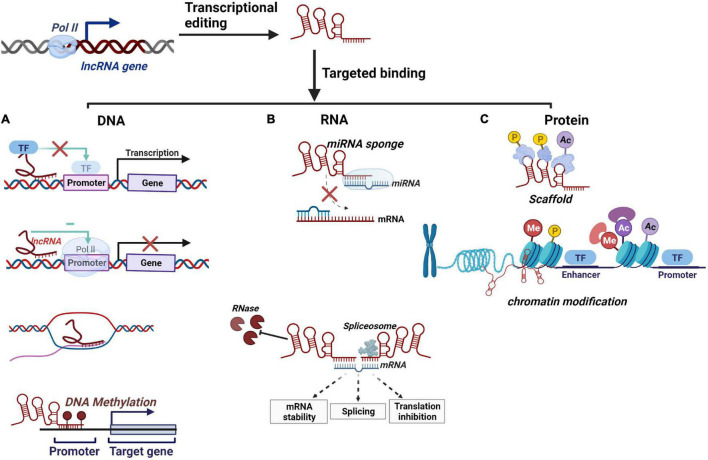
Schematic overview of lncRNAs’ molecular mechanisms. **(A)** lncRNAs regulate downstream gene transcription by binding to transcription factors, repression of RNA Pol II, complementary pairing with unstranded DNA bases, and DNA methylation. **(B)** lncRNAs serve as miRNA sponges to competitively bind with miRNA. lncRNAs regulate mRNA transcription by modulating mRNA stability and pre-mRNA splicing. lncRNAs can inhibit RNase degradation and promote mRNA stability. **(C)** lncRNAs can serve as a scaffold for protein phosphorylation and ubiquitination and regulate chromatin modification by employing histone-modifying complex.

A feature of lncRNAs is that they can generate a hybrid structure with DNA to influence gene expression. They mediate DNA methylation and transcriptional inhibition by complementary pairing with unstranded DNA bases. In addition, lncRNAs can inhibit the recruitment of Pol II or alter the binding of transcription factors at the promoter sequences whose function is blocked, modulating downstream target genes transcription (Figure 1A; Hainer and Martens, 2011; Schmitz et al., 2016). Recent findings suggest that the DNA-mediated innate immune response underpins antibacterial defense. In this regard, an investigation identified a multi-subunit complex built around HEXIM1 and lncRNA NEAT1, containing DNA-PK subunits and paraspeckle proteins, as a key nuclear regulator of DNA-mediated activation of innate immune response (Morchikh et al., 2017). Also, RNA–DNA complex formation has been proposed as an example of lncRNA–DNA interplay in mediating gene silencing or activation (Mondal et al., 2015; O’Leary et al., 2015). For instance, lncRNA PARTICLE, forming a DNA-lncRNA triplex, repress the tumor suppressor methionine adenosyltransferase (MAT2A) via methylation in response to irradiation, implicating it as a recruitment platform for gene-silencing machinery (O’Leary et al., 2015).

### RNA Level Regulation

At the RNA level, lncRNAs often function as lncRNA-miRNA-mRNA competing endogenous RNAs (ceRNAs) that serve as miRNA sponges and inhibit their regulatory effect on the target gene (Figure 1B; Salmena et al., 2011; Wang M. et al., 2019; Wang et al., 2020). For example, a ceRNA network constructed from pulmonary tuberculosis (PTB) patients suggested that lncRNAs regulate mRNAs expression may mediate by acting as sponged miRNAs (Zhang et al., 2020). Furthermore, severe acute respiratory syndrome coronavirus 2 (SARS-CoV-2), pandemic H1N1, and H7N9 infection induced upregulation of lncRNA-34087.27, which could serve as ceRNA, competitively binding with miR-302b-3p to stabilize IRF1 mRNA (Yang et al., 2022). Additionally, mRNA splicing is one of the biological functions that can be affected by lncRNAs through forming complementary double strands with transcripts (Figure 1B; De Troyer et al., 2020, p. 3). For instance, lncRNA MALAT1 can modulate the levels of serine/arginine (SR) proteins, thereby regulating the alternative splicing (AS) of pre-mRNA (Tripathi et al., 2010). Moreover, with lncRNA-mRNA dimer formation, lncRNAs can also inhibit RNase degradation and alter the stability and translation of cytoplasmic mRNAs (Figure 1B; Statello et al., 2021).

### Protein Level Regulation

Numerous lncRNAs localize on chromatin, interacting with proteins to facilitate or inhibit their binding and activity at targeted DNA regions. On the one hand, it can participate in protein phosphorylation by protein kinase or ubiquitin modification of a protein by ubiquitin-modified enzymes (Statello et al., 2021). For instance, HOTAIR is known to act at the posttranslational level by serving as an assembly scaffold for protein ubiquitination (Figure 1C). In addition, lncRNAs could regulate histone modification and chromatin accessibility, thereby regulating transcription (Figure 1C). A well-described example is the X chromosome dosage compensation process. The X-inactive-specific transcript (Xist) recruits polycomb repressive complex 2 (PRC2) and triggers large numbers of histones methylated as well as a cascade of events that entails chromosome remodeling to achieve stable silencing (Creamer and Lawrence, 2017; Jégu et al., 2017). Similarly, HOTAIR interacts with PRC2 favors epigenetic silencing through Enhance of Zeste2 (EZH2) catalyzed deposition of H3K27me3, facilitating the survival of virulent Mtb (Table 1; Subuddhi et al., 2020). Moreover, lncRNAs can be used as a protein structural component to affect protein spatial conformation.

**TABLE 1:** The regulatory role of lncRNAs in anti-TB immunity.

LncRNAs	Expression lever	Targets	Sample	Mtb strains	Effect	References
NEAT1	Up	miR-377-3p	Macrophages	H37Ra	Promote the expression of IL-6 and inhibit apoptosis	Sun et al., 2021
HOTAIR	Down	EZH2	Macrophages	H37Rv	Promote the SATB1 and DUSP4 transcript and inhibit the production of ROS	Subuddhi et al., 2020
Cox2	Up	NF-κB and Stat3	Macrophages	H37Ra	activate NF-κB and Stat3 to regulate inflammatory responses	Li D. et al., 2020
Cox2	Up	PERK/eIF2α/CHOP	Macrophages	BCG	Inhibit apoptosis and the accumulation of ROS	Xu et al., 2021
PACER	Up	Ptgs-2	Monocyte-Derived Macrophages	HN878 (clinical hypervirulent strain)	Promote ptgs2 transcription.	Tamgue et al., 2021
GAS5	Down	miR-18a-5p	Macrophages	H37Rv	Promote the cell viability and inflammatory response	Li et al., 2021
MEG3	Down	Not known	Macrophages	*M. bovis* BCG	Promote autophagy	Pawar et al., 2016
EPS	Down	JNK/MAPK	Macrophages	BCG	Attenuate apoptosis and promote autophagy	Ke et al., 2020
PCED1B-AS1	Down	miR-155	Macrophages	/	Attenuate apoptosis and promote autophagy	Li et al., 2019
MIAT	Up	miR-665	Macrophages	BCG	Attenuate autophagy and promote apoptosis	Jiang et al., 2021
CD244	Up	EZH2	CD8(+) T cells	/	Inhibit IFN-γ/TNF-α expression and promote Mtb proliferation	Wang et al., 2015
Lnc AC145676.2.1-6	Down	miR-29a	Whole blood	/	Interference with cytokine–cytokine receptor interactions and TLR signaling pathways.	Bai et al., 2019
Lnc-TGS1-1	Down	MiR-143	Whole blood	/	Thrombocytopenia and interference with the TLR signaling	Bai et al., 2019

## The Role of Long Non-Coding Rnas in Anti-Tuberculosis Immunity

**FIGURE 2:**
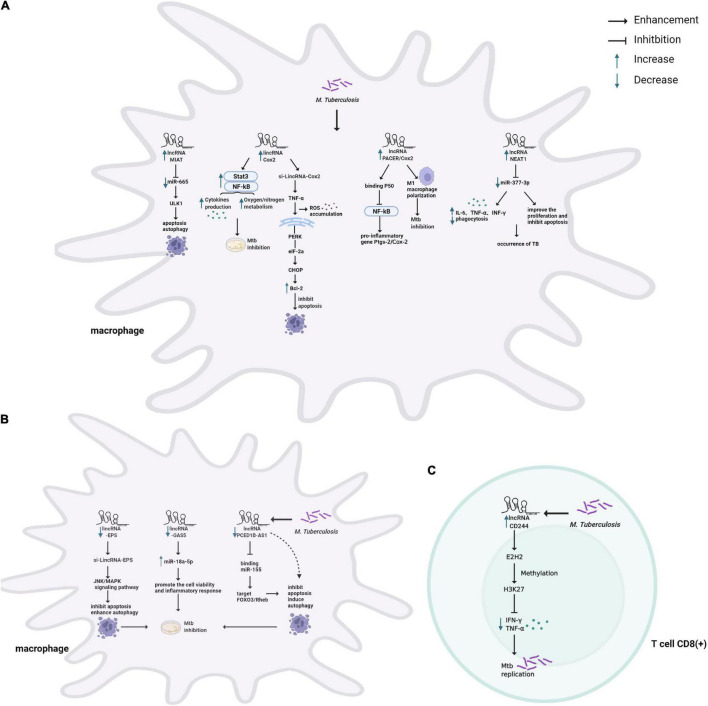
Schematic representation of the role of lncRNAs in host immune system during Mtb infection. **(A)** TB infection causes upregulation of lncRNAs modulating the immune system of macrophages. lncRNA MIAT modulates apoptosis and autophagy through the miR-665/ULK1 crosstalk. COX-2 modulates inflammatory response by activating NF-κb/STAT3, associating with p50 (a repressive subunit of NF-κb), and M1 macrophage polarization, and induces apoptosis by activating the PERK-eIF-2α-CHOP signaling pathway. NEAT1 modulates inflammatory response and apoptosis through targeting miR-377-3p. **(B)** TB infection causes downregulation of lncRNAs modulating the immune system of macrophages. LincRNA-EPS inhibited apoptosis and enhanced autophagy by activating the JNK/MAPK signaling pathway. GAS5 promotes the cell vitality and inflammatory response by sponging miR-18a-5p. PCED1B-AS1 can directly bind to miR-155 to inhibit apoptosis and induce autophagy. **(C)** TB infection causes upregulation of lncRNA-CD244 to interact with EZH2 and mediates H3K27 trimethylation at ifng/tnfa loci, thereby suppressing IFN-γ/TNF-α expression and increasing bacterial proliferation in T cells.

Different expression patterns of lncRNAs are emerging as critical regulators to control the function of innate and adaptive immune cell types and initiate effective defense mechanisms in TB (Figure 2 and Table 1; Chen Y. G. et al., 2017; Wei et al., 2021).

### The Role of Long Non-coding RNAs in Macrophages

Accumulating lines of evidence have revealed that lncRNAs play an important role in regulating the innate immune response of host macrophages, which are the sentinels to phagocytose and eliminate Mtb (Figure 2A,B). For one thing, lncRNAs participate in macrophage-mediated immune-inflammatory responses that an inducible program of inflammatory gene expression is central to anti-microbial defenses. For example, lincRNA-Cox2 mediates the activation/repression of immune genes that may activate NF-κB and STAT3 to regulate inflammatory responses for resistance of Mtb infection (Figure 2A; Carpenter et al., 2013; Li D. et al., 2020). In addition, lncRNA-PACER (also known as lncRNA-Cox2) was induced in Mtb infected macrophages, acting as a positive regulator of its proximal pro-inflammatory gene Ptgs-2 (also known as Cox2). Via mechanisms involving the sequestration of repressive NF-κB subunit p50 away from Ptgs-2 promoter (Krawczyk and Emerson, 2014; Tamgue et al., 2021). LncRNA-Cox2 can promote the activation of macrophages toward the pro-inflammatory M1 phenotype known to be efficient in killing Mtb (Figure 2A; Ye et al., 2018; Tamgue et al., 2021). Recently, some studies have found that in Mtb infected macrophages, the down-regulated lncRNA GAS5 might facilitate the cell vitality and the inflammatory response by sponging miR-18a-5p (Figure 2A; Li et al., 2021), and NEAT1 participates in inflammatory response through targeted regulation of miR-377-3p (Figure 2A; Sun et al., 2021).

For another, previous studies indicated that massive lncRNAs could regulate Mtb-induced apoptosis and autophagy of macrophages, playing a vital role in the pathogenesis of TB (Behar et al., 2011; Kim et al., 2019). For example, the Bacillus Calmette-Guerin (BCG)-infected macrophages induced apoptosis with upregulated lincRNA-Cox2 expression. Knockdown of Cox2 aggravated reactive oxygen species (ROS) accumulation and initiated apoptosis by activating the PERK-eIF-2α-CHOP signaling pathway (Figure 2B; Xu et al., 2021). A similar mechanism has been described for lncRNA-EPS, modulating apoptosis and autophagy by activating the JNK/MAPK signaling pathway in macrophages (Figure 2B; Ke et al., 2020). Additionally, down-regulation of lncRNA-MEG3 in infected macrophages induced autophagy and enhanced eradication of intracellular Mycobacterium Bovis BCG (Pawar et al., 2016). Recent studies have revealed that several lncRNAs act as ceRNAs to regulate apoptosis and autophagy. For example, lncRNA PCED1B-AS1 decreased in ATB patients compared to healthy individuals. The suppression of PCED1B-AS1 significantly attenuated apoptosis and enhanced autophagy in macrophages by sponging the miR-155 and inhibiting its targets FOXO3/Rheb (Figure 2B; Li et al., 2019). Moreover, lncRNA MIAT modulated macrophage apoptosis and autophagy upon BCG infection through the miR-665/ULK1 crosstalk (Figure 2A; Jiang et al., 2021).

### The Role of Long Non-coding RNAs in T Cells

Once the innate immune system is breached, the “human guardians,” including T and B cells, immediately enter a fighting state and initiate adaptive immunity. LncRNAs also regulate T cell-mediated immune regulation in TB (Figure 2C). CD4 + T cells play a dominant role in the host immune response of TB (Jasenosky et al., 2015). The study analyzed the lncRNA profile in CD4 + T cells and revealed that compared with healthy controls, lncRNAs showed abnormal expression in ATB and LTBI (Yi et al., 2014). In addition, significantly enriched signaling pathways based on deregulated mRNAs were cytokine-cytokine receptor interaction, mitogen-activated protein kinase (MAPK), and TLR signaling pathway (Yi et al., 2014).

In general, CD8 + T cells were previously thought to be less critical than CD4 + T cells in the immune response to TB. It has been newly stated its non-redundant role with a specific CD8 + T cell response (Lin and Flynn, 2015). A recent study confirmed that lncRNA-CD244 epigenetically repressed the IFN-γ and TNF-α expression in CD8 + T cells (Figure 2C; Wang et al., 2015). Adoptive transfer of CD244–depressed CD8 + T cells to Mtb-infected mice reduced infection and pathology compared to mice transplanted with wild-type CD8 + cells (Wang et al., 2015). Moreover, Heme Oxygenase 1 (HMOX1) was increased after Mtb infection and can distinguish LTBI from ATB in the previous research (Costa et al., 2016). Also, 328 differentially expressed lncRNAs were found in CD8 + T cells’ response to ATB. Among them, lincRNA XLOC_014219 was upregulated, while its nearby protein-coding gene HMOX1 was significantly decreased. It is essential to uncover why HMOX1 is downregulated and whether lincRNA XLOC_014219 relates to it, which is ultimately involved in the dysfunction of CD8 + T cells (Fu et al., 2017a).

### The Role of Long Non-coding RNAs in B Cells

It has come to light that lncRNAs can influence antibodies produced by B cells and impact B cell biology by regulating survival signals during activation (Zeni and Mraz, 2021). For instance, a study reported that 844 lncRNAs differentially expressed in B cell samples. Additionally, SOCS3 is an essential negative regulator of cytokine response to Mtb infection, and its upstream lncRNA XLOC_012582 highly increased in B cells of ATB. Whether upregulation of XLOC_012582 leads to overexpression of SOCS3 and ultimately participates in the progression of TB needs in-depth investigation (Fu et al., 2017b). Findings provided new insight into the pathogenesis of TB. However, discoveries related to B cells are like a tip of an iceberg.

In summary, the immune protection mechanism of TB is complex and comprehensive. Although there have been preliminary studies on the role of lncRNAs in different host cells to Mtb, their specific functions in immunity are mainly unexplored.

## Long Non-Coding Rnas as Diagnosis Biomarkers in Tuberculosis

So far, most clinical diagnosis methods have inherent limitations (Pai et al., 2016). Methods like smear microscopy and mycobacterium culture have insufficient sensitivity and timeliness and have a poor detection rate of smear-negative PTB (Walzl et al., 2011; Fang et al., 2021; Mirzaei et al., 2021), while interferon-gamma release assays (IGRA) unable to discriminate between LTBI and ATB (Walzl et al., 2018). The recent recommendations of the WHO include non-pathogen-based detection to improve the identification of clinically diagnosed TB with rapid and universal methods (Martinez and Andrews, 2019). Therefore, biomarkers of the host immune responses might provide critical insights to solve this problem. Some ncRNAs are considered to be biomarkers (Beermann et al., 2016), such as miR-889 targets that can be manipulated for antimycobacterial therapeutic purposes and candidate biomarkers for LTBI (Chen et al., 2020). Relative to investigated in high detail miRNAs, the roles of lncRNAs remain largely elusive (Lee, 2012). Nevertheless, a rising number of dysregulation processes indicate that lncRNAs are highly promising as biomarkers of TB (Table 2).

**TABLE 2:** Overview of the candidate lncRNA biomarkers in TB.

Analysis	Candidates of lncRNA biomarkers	Sample	Effect	References
PBMCs	Upregulated (MIR3945HG V1 and MIR3945HG V2) in PTB patients	Active PTB patients and healthy donors vaccinated with BCG	Promising candidate diagnostic markers for TB	Yang et al., 2016
PBMCs	NEAT1 (both NEAT1_1 and NEAT1_2) declined gradually with treatment	TB patients and healthy group	Potential indicator for patient prognosis of TB	Huang et al., 2018
PBMCs	Downregulated (PCED1B-AS1) in ATB patients	ATB patients and healthy individuals	May represent a novel early diagnostic marker of ATB	Li et al., 2019
PBMCs	Downregulated (n344917) in clinically diagnosed PTB	clinically diagnosed PTB, PTB with an etiological evidence and non-TB disease controls	Potential molecular biomarker for the clinically diagnosed PTB	Meng et al., 2021
Plasma	Upregulated (ENST00000354432, ENST00000427151) in TB patients	TB patients, community acquired pneumonia and healthy individuals	Potential molecular biomarkers for the rapid diagnosis of TB	He et al., 2017
Sputum and plasma	Upregulated LOC152742 in ATB patients	ATB patients, obsolete TB patients, individuals affected with BCG, and normal individuals,	Potential biomarker for diagnosis and therapy of ATB	Wang L. et al., 2019
Plasma	Differently expressed (lncRNAs uc.48 + and NR_105053) between the untreated and the cured TB	Untreated TB and cured TB subjects	Potential biomarkers to distinguish between untreated and cured TB, provide an experimental basis to evaluate the effect of TB treatment	Li Z.-B. et al., 2020
Plasma	Upregulated (NR_038221, NR_003142, and ENST00000570366), downregulated (ENST00000422183)	ATB patients and healthy control	Potential biomarkers for early diagnosis of TB	Chen Z.-L. et al., 2017
Serum exosomes	Downregulated (NON-HSAT101518.2, NON-HSAT067134.2, NON-HSAT148822.1 NON-HSAT078957.2) in ATB patients	ATB patients and healthy individuals	Discriminate ATB from healthy individuals	Fang et al., 2021
PBMC	Differently expressed (ENST00000497872, n333737, and n335265) in PTB and healthy control	Clinically diagnosed PTB, microbiologically confirmed PTB cases, non-TB disease controls, healthy subjects	Facilitate the early identification of PTB cases among suspected patients with negative Mtb microbiological evidence	Hu et al., 2020
Lung tissue	Upregulated (ENST00000429730.1 and MSTRG.93125.4)	sputum-negative pulmonary TB patients	Potential indicators of metabolic activity in TB lesions for sputum-negative tuberculosis	Wang et al., 2021
Whole blood	AC079767.4	TB patients and healthy individuals	AC079767.4 polymorphisms may potentially act as biomarkers for TB diagnostic and even as therapeutic targets	Zhao et al., 2017
Whole blood	Downregulated (AC145676.2.1-6 and TGS1-1) in TB patients	TB patients and healthy individuals	TGS1-1 and its variants 4737420 may be predictive indicators of anti-TB drug-induced adverse drug reactions	Bai et al., 2019
Whole blood	RIPK2	TB patients and healthy control	Might serve as a hazard for TB in this Western Chinese Han population	Song et al., 2019a
Peripheral blood	HNF1B-3:1	TB cases and healthy subjects	May influence the clinical manifestations of TB	Wu et al., 2020
Whole blood	RP11-37B2.1	TB cases and healthy subjects	Potential biosignatures for thrombocytopenia during anti-TB treatment	Song et al., 2019b
Whole blood	CASC8	TB patients and healthy individuals	Biomarker for the progression of clinical TB	Liu et al., 2020

### Biomarkers in Peripheral Blood Mononuclear Cells

Accumulating lines of studies have revealed the abnormally expressed lncRNAs in peripheral blood mononuclear cells (PBMCs) of TB patients (Table 2). A study reported that two significantly aberrantly expressed lncRNA (MIR3945HG V1 and MIR3945HG V2) in PBMCs samples from active PTB patients have the potential to be novel diagnostic biomarkers (Yang et al., 2016). Furthermore, the expression of NEAT1 (both NEAT1_1 and NEAT1_2) in TB patients was higher than healthy control, declined gradually with treatment, and was restored to the normal level. This dynamic change could reflect the efficacy of anti-TB therapy. Therefore, NEAT1 may serve as a potential indicator for patient prognosis of TB (Huang et al., 2018). Additionally, the study suggested that downregulated PCED1B-AS1 in PBMCs and THP-1 show promise as a new early diagnostic biomarker for ATB (Li et al., 2019). Nevertheless, little information has been done on the underlying mechanisms of lncRNAs above. To enhance the PTB identification, lncRNA n344917 was confirmed down-regulated in PBMC of PTB. Hence, a web-based prediction model combining the molecular biomarker n344917, laboratory, and EHR variables was constructed and could serve as a user-friendly, accurate platform to improve the clinical diagnosis of PTB (Meng et al., 2021).

### Biomarkers in Plasma or Serum

Differentially expressed lncRNAs in the plasma or serum of TB patients have also been explored as potential diagnostic biomarkers (Table 2). For instance, expression levels of ENST00000354432 and ENST00000427151 were suggested to act as biomarkers for the early detection of TB (He et al., 2017). LOC152742 in plasma had higher specificity in ATB and gradually downregulated in the treatment. Hence it could serve as a novel biomarker for the diagnosis and therapy of ATB (Wang L. et al., 2019). Also, plasma lncRNAs might act as potential biomarkers to evaluate TB cure in an efficient and precise manner. LncRNAs uc.48 + and NR_105053 may serve as biomarkers to distinguish between untreated TB patients and cured TB subjects (Li Z.-B. et al., 2020). Recently, studies reported some lncRNA sets with high diagnostic sensitivity and specificity. For example, ceRNA analysis of four differentially expressed lncRNAs (NR_038221, NR_003142, ENST00000570366, and ENST00000422183) was demonstrated to discriminate PTB from healthy individuals. The results showed that NR_038221 was the most significantly associated with TB (Chen Z.-L. et al., 2017). Their previous study had verified hsa-miR-378a-3p as a potential biomarker for PTB, which was associated with NR_038221, indicating that NR_038221 and hsa-miR-378a-3p might play a similar function during the biological process of PTB (Zhang et al., 2013; Chen Z.-L. et al., 2017). At present, the development of high-throughput experimental technologies has led to a rapid expansion of lncRNA research. For example, an integrated analysis of the GEO dataset and the NON-CODE database identified four significantly downregulated lncRNAs (NON-HSAT101518.2, NON-HSAT067134.2, NON-HSAT148822.1, and NON-HSAT078957.2) in serum exosomes of ATB patients. ROC curve analysis suggests that these four lncRNAs can discriminate ATB from healthy individuals with high specificity and sensitivity (Fang et al., 2021).

### Biomarkers in Bacteria-Negative Tuberculosis

Accurate diagnosis of complete inactivation of TB lesions remains a challenge concerning sputum-negative TB, one of the significant factors for the development and spread of ATB (Horsburgh, 2004). Clinically diagnosed PTB patients without microbiological evidence of Mtb often lead to misdiagnosis or delayed diagnosis. Therefore, A study validated the lncRNAs and corresponding predictive models to effectively diagnose these patients, finding differentially expressed lncRNAs (ENST00000497872, n333737, n335265) between PTB patients and the healthy group. These lncRNAs might be used as putative biomarkers to facilitate the early identification of PTB cases among suspected patients with negative microbiological evidence (Table 2; Hu et al., 2020). In addition, lncRNAs (ENST00000429730.1 and MSTRG.93125.4) were upregulated in lung tissues with high metabolic activity demonstrated by fluorine-18-fluorodeoxyglucose positron emission tomography/computed tomography from sputum-negative TB patients compared with low metabolic activity. Two lncRNAs might be considered potential metabolic activity indicators in TB lesions for sputum-negative TB (Table 2; Wang et al., 2021).

### Long Non-coding RNA Polymorphism and Tuberculosis Susceptibility

Abundant evidence from the investigations suggests that host genetic factors contribute to determine susceptibility to TB disease (Möller and Hoal, 2010). Recent advances in lncRNA research have preliminarily explored the relationship between lncRNA single nucleotide polymorphisms (SNPs), TB susceptibility, and clinical manifestations in TB patients of the western Chinese Han population (Table 2). For example, a study uncovered that the lncRNA AC079767.4 might be involved in the progression of TB infection. In addition, the C allele of SNP rs12477677 in AC079767.4 was associated with reduced susceptibility to PTB (Zhao et al., 2017). Moreover, the SNPs (rs12477677 and rs1055229) may influence clinical TB disease. Conceivably, AC079767.4 polymorphisms may serve as novel biomarkers for TB diagnostic and even as therapeutic targets (Zhao et al., 2017). Similarly, the potential TB-associated promoting effects were identified for the decreased expression levels of lnc-AC145676.2.1-6 and lnc-TGS-1 (Bai et al., 2019). Recently, the SNPs of rs39509 G allele in PIPK2 Near gene-3 region, lncRNA - HNF1B - 3:1, lncRNA - RP11-37 b2. 1, lncRNA - CASC8 has also been proved to be associated with TB susceptibility (Song et al., 2019a,b; Liu et al., 2020; Wu et al., 2020). These lncRNA polymorphisms are promising molecular biomarkers for clinical TB infection and/or efficacy evaluation. However, there are some questions need to be answered, such as are there sex and ethnic differences in differentially expressed lncRNAs?

## Discussion

Overall, the advancement of sequencing technology has facilitated the discovery of lncRNAs with unknown functions. These lncRNAs can thwart disease or lead to disease progression. Importantly, lncRNAs have presented a diverse perspective on the regulation of TB, especially complicated immune regulation for multiple biomolecular interactions. By high-throughput sequencing and deepening validation, lncRNAs have been found as potential biomarkers for diagnosis, monitoring progression, and clinical efficacy of TB. However, the complexity of the lncRNAs themselves and the lack of accurate databases for the lncRNAs discovered are restricting research to the single analysis of differences in gene expression. We are still far from completely understanding how lncRNAs influence complex physiopathological processes of TB, and several aspects of lncRNA remain enigmatic. For instance, whether lncRNA regulates Mtb infection as an infection phenomenon or a specific bacterial infection phenomenon under certain conditions. In addition, there is a lack of using lncRNAs as biomarkers to indicate progression from latent infections to clinical disease. Despite the questions, lncRNAs are highly ideal biomarkers. Further validation studies on different ethnic populations and function experiments in a large-scale cohort help to confirm the roles of the lncRNAs, and may also help identify biomarkers for latent infections. Most of the research on ncRNAs mainly focuses on miRNA, but understanding the lncRNA function can only be achieved from more studies on a case-by-case basis.

Several new biotechnologies such as Xpert MTB/RIF Ultra have been developed in this context. It has increased sensitivity for diagnosis but is sophisticated. More accurate, rapid, and cost-effective tests are needed to improve TB detection. Various platforms have explored other possible biomarkers, such as host marker signatures, including host gene expression, protein, and metabolites. Some have combined omics techniques which have made some progress in distinguishing LTBI from ATB to explore progression to TB. However, such measurement of expression of markers requires substantial laboratory infrastructure and is time-consuming. Additionally, the most work done so far has been small case-control studies, and the diagnostic potential of these markers still has to be confirmed. The continued study of TB-associated lncRNAs will reveal more unanticipated biomarkers for predicting progression, response to treatment, and relapse. Moreover, the personalized lncRNA-targeted drugs in host-directed therapy (HDTS) are under development.

## Author Contributions

XZ collected the related manuscript and drafted the manuscript. XZ, CC, and YX discussed and developed its conceptual framework. CC and YX proofread the manuscript. YX and XZ revised the review. All authors contributed to the article and approved the submitted version.

## Conflict of Interest

The authors declare that the research was conducted in the absence of any commercial or financial relationships that could be construed as a potential conflict of interest.

## Publisher’s Note

All claims expressed in this article are solely those of the authors and do not necessarily represent those of their affiliated organizations, or those of the publisher, the editors and the reviewers. Any product that may be evaluated in this article, or claim that may be made by its manufacturer, is not guaranteed or endorsed by the publisher.

## References

[B1] Bai H. Wu Q. Hu X. Wu T. Song J. Liu T. (2019). ‘Clinical significance of lnc-AC145676.2.1-6 and lnc-TGS1-1 and their variants in western Chinese tuberculosis patients’. *Int. J. Infect. Dis.* 84 8–14. 10.1016/j.ijid.2019.04.018 31028876

[B2] Beermann J. Piccoli M.-T. Viereck J. Thum T. (2016). ‘Non-coding RNAs in development and disease: background. mechanisms, and therapeutic approaches’, *Physiol. Rev.* 96 1297–1325. 10.1152/physrev.00041.2015 27535639

[B3] Behar S. M. Martin C. J. Booty M. G. Nishimura T. Zhao X. Gan H.-X. (2011). ‘Apoptosis is an innate defense function of macrophages against mycobacterium tuberculosis’. *Mucosal Immunol.* 4 279–287. 10.1038/mi.2011.3 21307848 PMC3155700

[B4] Carpenter S. Aiello D. Atianand M. K. Ricci E. P. Gandhi P. Hall L. L. (2013). ‘A long noncoding RNA mediates both activation and repression of immune response genes’. *Science* 341 789–792. 10.1126/science.1240925 23907535 PMC4376668

[B5] Chen D.-Y. Chen Y.-M. Lin C.-F. Lo C.-M. Liu H.-J. Liao T.-L. (2020). ‘MicroRNA-889 inhibits autophagy to maintain mycobacterial survival in patients with latent tuberculosis infection by targeting TWEAK’. *mBio* 11 e3045–e3019. 10.1128/mBio.03045-19 31992621 PMC6989109

[B6] Chen Y. G. Satpathy A. T. Chang H. Y. (2017). ‘Gene regulation in the immune system by long noncoding RNAs’. *Nat. Immunol.* 18 962–972. 10.1038/ni.3771 28829444 PMC9830650

[B7] Chen Z.-L. Wei L.-L. Shi L.-Y. Li M. Jiang T.-T. Chen J. (2017). ‘Screening and identification of lncRNAs as potential biomarkers for pulmonary tuberculosis’. *Sci. Rep.* 7:16751. 10.1038/s41598-017-17146-y 29196714 PMC5711916

[B8] Costa D. L. Namasivayam S. Amaral E. P. Arora K. Chao A. Mittereder L. R. (2016). ‘Pharmacological inhibition of host heme oxygenase-1 suppresses mycobacterium tuberculosis infection in vivo by a mechanism dependent on T lymphocytes’. *mBio* 7 e1675–e1616. 10.1128/mBio.01675-16 27795400 PMC5080384

[B9] Creamer K. M. Lawrence J. B. (2017). ‘XIST RNA: a window into the broader role of RNA in nuclear chromosome architecture’. *Philos. Trans. R. Soc. Lond. B. Biol. Sci.* 372:20160360. 10.1098/rstb.2016.0360 28947659 PMC5627162

[B10] De Troyer L. Zhao P. Pastor T. Baietti M. F. Barra J. Vendramin R. (2020). ‘Stress-induced lncRNA LASTR fosters cancer cell fitness by regulating the activity of the U4/U6 recycling factor SART3’. *Nucleic Acids Res.* 48 2502–2517. 10.1093/nar/gkz1237 31956895 PMC7049684

[B11] Fang Y. Zhao J. Wang X. Wang X. Wang L. Liu L. (2021). Identification of differentially expressed lncRNAs as potential plasma biomarkers for active tuberculosis. *Tuberculosis* 128:102065. 10.1016/j.tube.2021.102065 33690081

[B12] Fathizadeh H. Hayat S. M. G. Dao S. Ganbarov K. Tanomand A. Asgharzadeh M. (2020). ‘Long non-coding RNA molecules in tuberculosis’. *Int. J. Biol. Macromol.* 156 340–346. 10.1016/j.ijbiomac.2020.04.030 32283111

[B13] Fu Y. Gao K. Tao E. Li R. Yi Z. (2017a). ‘Aberrantly expressed long non-coding RNAs in CD8+ T cells response to active tuberculosis’. *J. Cell. Mol. Biochem.* 118 4275–4284. 10.1002/jcb.26078 28422321

[B14] Fu Y. Xu X. Xue J. Duan W. Yi Z. (2017b). ‘Deregulated lncRNAs in B cells from patients with active tuberculosis’. *PLoS One* 12:e0170712. 10.1371/journal.pone.0170712 28125665 PMC5268381

[B15] Guo C.-J. Ma X.-K. Xing Y.-H. Zheng C.-C. Xu Y.-F. Shan L. (2020). Distinct processing of lncRNAs contributes to non-conserved functions in stem cells. *Cell* 181 621.e–636.e. 10.1016/j.cell.2020.03.006 32259487

[B16] Hainer S. J. Martens J. A. (2011). ‘Transcription of ncDNA: many roads lead to local gene regulation’. *Transcription* 2 120–123. 10.4161/trns.2.3.15684 21826282 PMC3149688

[B17] He J. Ou Q. Liu C. Shi L. Zhao C. Xu Y. (2017). ‘Differential expression of long non-coding RNAs in patients with tuberculosis infection’. *Tuberculosis* 107 73–79. 10.1016/j.tube.2017.08.007 29050775

[B18] Heydarnezhad Asl M. Pasban Khelejani F. Bahojb Mahdavi S. Z. Emrahi L. Jebelli A. Mokhtarzadeh A. (2022). The various regulatory functions of long noncoding RNAs in apoptosis, cell cycle, and cellular senescence. *J. Cell. Biochem.* [Preprint] 10.1002/jcb.30221 35106829

[B19] Horsburgh C. R. (2004). ‘Priorities for the treatment of latent tuberculosis infection in the United States’. *N. Engl. J. Med.* 350 2060–2067. 10.1056/NEJMsa031667 15141044

[B20] Hu X. Liao S. Bai H. Gupta S. Zhou Y. Zhou J. (2020). ‘Long noncoding RNA and predictive model to improve diagnosis of clinically diagnosed pulmonary tuberculosis’. *J. Clin. Microbiol.* 58 e1973–e1919. 10.1128/JCM.01973-19 32295893 PMC7315016

[B21] Huang J.-Z. Chen M. Chen D. Gao X.-C. Zhu S. Huang H. (2017). A peptide encoded by a putative lncRNA HOXB-AS3 suppresses colon cancer growth. *Mol. Cell* 68 171.e–184.e. 10.1016/j.molcel.2017.09.015 28985503

[B22] Huang S. Huang Z. Luo Q. Qing C. (2018). ‘The Expression of lncRNA NEAT1 in human tuberculosis and its antituberculosis effect’. *Biomed. Res. Int.* 2018:9529072. 10.1155/2018/9529072 30534569 PMC6252192

[B23] Jasenosky L. D. Scriba T. J. Hanekom W. A. Goldfeld A. E. (2015). ‘T cells and adaptive immunity to mycobacterium tuberculosis in humans’. *Immunol. Rev.* 264 74–87. 10.1111/imr.12274 25703553

[B24] Jégu T. Aeby E. Lee J. T. (2017). ‘The X chromosome in space’. *Nat. Rev. Genet.* 18 377–389. 10.1038/nrg.2017.17 28479596

[B25] Jiang F. Lou J. Zheng X.-M. Yang X.-Y. (2021). ‘LncRNA MIAT regulates autophagy and apoptosis of macrophage infected by mycobacterium tuberculosis through the miR-665/ULK1 signaling axis’. *Mol. Immunol.* 139 42–49. 10.1016/j.molimm.2021.07.023 34454184

[B26] Kazemzadeh M. Safaralizadeh R. Orang A. V. (2015). ‘LncRNAs: emerging players in gene regulation and disease pathogenesis’. *J. Genet.* 94 771–784. 10.1007/s12041-015-0561-6 26690535

[B27] Ke Z. Lu J. Zhu J. Yang Z. Jin Z. Yuan L. (2020). ‘Down-regulation of lincRNA-EPS regulates apoptosis and autophagy in BCG-infected RAW264.7 macrophages via JNK/MAPK signaling pathway’. *Infect. Genet. Evol.* 77:104077. 10.1016/j.meegid.2019.104077 31669366

[B28] Kim M.-S. Pinto S. M. Getnet D. Nirujogi R. S. Manda S. S. Chaerkady R. (2014). ‘A draft map of the human proteome’. *Nature* 509 575–581. 10.1038/nature13302 24870542 PMC4403737

[B29] Kim Y. S. Silwal P. Kim S. Y. Yoshimori T. Jo E.-K. (2019). ‘Autophagy-activating strategies to promote innate defense against mycobacteria’. *Exp. Mol. Med.* 51 1–10. 10.1038/s12276-019-0290-7 31827065 PMC6906292

[B30] Krawczyk M. Emerson B. M. (2014). ‘p50-associated COX-2 extragenic RNA (PACER) activates COX-2 gene expression by occluding repressive NF-κB complexes’. *Elife* 3:e01776. 10.7554/eLife.01776 24843008 PMC4017649

[B31] Lee J. T. (2012). ‘Epigenetic regulation by long noncoding RNAs. *Science* 338 1435–1439. 10.1126/science.1231776 23239728

[B32] Li D. Gao C. Zhao L. Zhang Y. (2020). ‘Inflammatory response is modulated by lincRNACox2 via the NF−κB pathway in macrophages infected by mycobacterium tuberculosis’. *Mol. Med. Rep.* 21 2513–2521. 10.3892/mmr.2020.11053 32323851 PMC7185307

[B33] Li M. Cui J. Niu W. Huang J. Feng T. Sun B. (2019). ‘Long non-coding PCED1B-AS1 regulates macrophage apoptosis and autophagy by sponging miR-155 in active tuberculosis’. *Biochem. Biophys. Res. Commun.* 509 803–809. 10.1016/j.bbrc.2019.01.005 30621915

[B34] Li Y. Sun L. Liu J. Xu G. Hu Y. Qin A. (2021). ‘Down-regulation of GAS5 has diagnostic value for tuberculosis and regulates the inflammatory response in mycobacterium tuberculosis infected THP-1 cells’. *Tuberculosis* 132:102141. 10.1016/j.tube.2021.102141 34808575

[B35] Li Z.-B. Han Y.-S. Wei L.-L. Shi L.-Y. Yi W.-J. Chen J. (2020). Screening and identification of plasma lncRNAs uc.48+ and NR_105053 as potential novel biomarkers for cured pulmonary tuberculosis. *Int. J. Infect. Dis.* 92, 141–150. 10.1016/j.ijid.2020.01.005 31931167

[B36] Lin P. L. Flynn J. L. (2015). ‘CD8 T cells and mycobacterium tuberculosis infection’. *Semin. Immunopathol.* 37 239–249. 10.1007/s00281-015-0490-8 25917388 PMC4439333

[B37] Liu G. Xia R. Wang Q. Wang Z. Ying B. Yan H. (2020). ‘Significance of LncRNA CASC8 genetic polymorphisms on the tuberculosis susceptibility in Chinese population’. *J. Clin. Lab. Anal.* 34:e23234. 10.1002/jcla.23234 32034808 PMC7307370

[B38] Lyu M. Cheng Y. Zhou J. Chong W. Wang Y. Xu W. (2021). ‘Systematic evaluation, verification and comparison of tuberculosis-related non-coding RNA diagnostic panels’. *J. Cell. Mol. Med.* 25 184–202. 10.1111/jcmm.15903 33314695 PMC7810967

[B39] Martinez L. Andrews J. R. (2019). ‘Improving tuberculosis case finding in persons living with advanced HIV through new diagnostic algorithms’. *Am. J. Respir. Crit. Care Med.* 199 559–560. 10.1164/rccm.201809-1702ED 30273498 PMC6396861

[B40] McCaffrey E. F. Donato M. Keren L. Chen Z. Delmastro A. Fitzpatrick M. B. (2022). ‘The immunoregulatory landscape of human tuberculosis granulomas’. *Nat. Immunol.* 23 318–329. 10.1038/s41590-021-01121-x 35058616 PMC8810384

[B41] McFadden E. J. Hargrove A. E. (2016). ‘Biochemical methods to investigate lncRNA and the influence of lncRNA:Protein complexes on chromatin’. *Biochemistry* 55 1615–1630. 10.1021/acs.biochem.5b01141 26859437 PMC5010801

[B42] Medley J. Goff A. Bettencourt P. J. G. Dare M. Cole L. Cantillon D. (2022). ‘Dissecting the mycobacterium bovis BCG response to macrophage infection to help prioritize targets for anti-tuberculosis drug and vaccine discovery’. *Vaccines* 10:113. 10.3390/vaccines10010113 35062774 PMC8780277

[B43] Meng Z. Wang M. Guo S. Zhou Y. Lyu M. Hu X. (2021). ‘Novel long non-coding RNA and LASSO prediction model to better identify pulmonary tuberculosis: a case-control study in China’. *Front. Mol. Biosci.* 8:632185. 10.3389/fmolb.2021.632185 34113649 PMC8185277

[B44] Mirzaei R. Babakhani S. Ajorloo P. Ahmadi R. H. Hosseini-Fard S. R. Keyvani H. (2021). ‘The emerging role of exosomal miRNAs as a diagnostic and therapeutic biomarker in mycobacterium tuberculosis infection’. *Mol. Med.* 27:34. 10.1186/s10020-021-00296-1 33794771 PMC8017856

[B45] Möller M. Hoal E. G. (2010). ‘Current findings, challenges and novel approaches in human genetic susceptibility to tuberculosis’. *Tuberculosis* 90 71–83. 10.1016/j.tube.2010.02.002 20206579

[B46] Mondal T. Subhash S. Vaid R. Enroth S. Uday S. Reinius B. (2015). ‘MEG3 long noncoding RNA regulates the TGF-β pathway genes through formation of RNA-DNA triplex structures’. *Nat. Commun.* 6:7743. 10.1038/ncomms8743 26205790 PMC4525211

[B47] Morchikh M. Cribier A. Raffel R. Amraoui S. Cau J. Severac D. (2017). HEXIM1 and NEAT1 long non-coding RNA form a multi-subunit complex that regulates DNA-mediated innate immune response. *Mol. Cell* 67 387.e–399.e. 10.1016/j.molcel.2017.06.020 28712728

[B48] O’Leary V. B. Ovsepian S. V. Carrascosa L. G. Buske F. A. Radulovic V. Niyazi M. (2015). ‘PARTICLE, a triplex-forming long ncRNA, Regulates locus-specific methylation in response to low-dose irradiation’. *Cell Rep.* 11 474–485. 10.1016/j.celrep.2015.03.043 25900080

[B49] Pai M. Behr M. A. Dowdy D. Dheda K. Divangahi M. Boehme C. C. (2016). ‘Tuberculosis’. *Nat. Rev. Dis. Primers* 2:16076. 10.1038/nrdp.2016.76 27784885

[B50] Pang K. C. Frith M. C. Mattick J. S. (2006). ‘Rapid evolution of noncoding RNAs: lack of conservation does not mean lack of function’. *Trends Genet.* 22 1–5. 10.1016/j.tig.2005.10.003 16290135

[B51] Pawar K. Hanisch C. Palma Vera S. E. Einspanier R. Sharbati S. (2016). ‘Down regulated lncRNA MEG3 eliminates mycobacteria in macrophages via autophagy’. *Sci. Rep.* 6:19416. 10.1038/srep19416 26757825 PMC4725832

[B52] Quinn J. J. Chang H. Y. (2016). ‘Unique features of long non-coding RNA biogenesis and function’. *Nat. Rev. Genet.* 17 47–62. 10.1038/nrg.2015.10 26666209

[B53] Salmena L. Poliseno L. Tay Y. Kats L. Pandolfi P. P. (2011). ‘A ceRNA hypothesis: the rosetta stone of a hidden RNA language?’. *Cell* 146 353–358. 10.1016/j.cell.2011.07.014 21802130 PMC3235919

[B54] Schmitz S. U. Grote P. Herrmann B. G. (2016). ‘Mechanisms of long noncoding RNA function in development and disease’. *Cell. Mol. Life Sci.* 73 2491–2509. 10.1007/s00018-016-2174-5 27007508 PMC4894931

[B55] Song J. Liu T. Jiao L. Zhao Z. Hu X. Wu Q. (2019a). ‘RIPK2 polymorphisms and susceptibility to tuberculosis in a Western Chinese Han population’. *Infect. Genet. Evol.* 75:103950. 10.1016/j.meegid.2019.103950 31279003

[B56] Song J. Liu T. Zhao Z. Hu X. Wu Q. Peng W. (2019b). ‘Genetic polymorphisms of long noncoding RNA RP11-37B2.1 associate with susceptibility of tuberculosis and adverse events of antituberculosis drugs in west China’. *J. Clin. Lab. Anal.* 33:e22880. 10.1002/jcla.22880 30924187 PMC6595342

[B57] Stanley S. A. Cox J. S. (2013). ‘Host-pathogen interactions during mycobacterium tuberculosis infections’. *Curr. Top. Microbiol. Immunol.* 374 211–241. 10.1007/82_2013_332 23881288

[B58] Statello L. Guo C.-J. Chen L.-L. Huarte M. (2021). ‘Gene regulation by long non-coding RNAs and its biological functions’. *Nat. Rev. Mol. Cell. Biol.* 22 96–118. 10.1038/s41580-020-00315-9 33353982 PMC7754182

[B59] Subuddhi A. Kumar M. Majumder D. Sarkar A. Ghosh Z. Vasudevan M. (2020). ‘Unraveling the role of H3K4 trimethylation and lncRNA HOTAIR in SATB1 and DUSP4-dependent survival of virulent mycobacterium tuberculosis in macrophages’. *Tuberculosis* 120:101897. 10.1016/j.tube.2019.101897 32090865

[B60] Sun Q. Shen X. Ma J. Lou H. Sha W. (2021). LncRNA NEAT1 participates in inflammatory response in macrophages infected by mycobacterium tuberculosis through targeted regulation of miR-377-3p. *Microb. Pathog.* 150:104674. 10.1016/j.micpath.2020.104674 33271233

[B61] Tamgue O. Chia J. E. Brombacher F. (2021). ‘Triptolide modulates the expression of inflammation-associated lncRNA-PACER and lincRNA-p21 in mycobacterium tuberculosis-infected monocyte-derived macrophages’. *Front. Pharmacol.* 12:618462. 10.3389/fphar.2021.618462 33912039 PMC8071990

[B62] Tripathi V. Ellis J. D. Shen Z. Song D. Y. Pan Q. Watt A. T. (2010). ‘The nuclear-retained noncoding RNA MALAT1 regulates alternative splicing by modulating SR splicing factor phosphorylation’. *Mol. Cell* 39 925–938. 10.1016/j.molcel.2010.08.011 20797886 PMC4158944

[B63] Ulitsky I. Bartel D. P. (2013). ‘lincRNAs: genomics, evolution, and mechanisms’. *Cell* 154 26–46. 10.1016/j.cell.2013.06.020 23827673 PMC3924787

[B64] WHO (2021). *Global Tuberculosis Report 2021* Available Online at: https://www.who.int/teams/global-tuberculosis-programme/tb-reports/global-tuberculosis-report-2021 (accessed October 30, 2021).

[B65] Walzl G. McNerney R. du Plessis N. Bates M. McHugh T. D. Chegou N. N. (2018). ‘Tuberculosis: advances and challenges in development of new diagnostics and biomarkers’. *Lancet Infect. Dis.* 18 e199–e210. 10.1016/S1473-3099(18)30111-7 29580818

[B66] Walzl G. Ronacher K. Hanekom W. Scriba T. J. Zumla A. (2011). ‘Immunological biomarkers of tuberculosis’. *Nat. Rev. Immunol.* 11 343–354. 10.1038/nri2960 21475309

[B67] Wang K. C. Chang H. Y. (2011). ‘Molecular mechanisms of long noncoding RNAs’. *Mol. Cell* 43 904–914. 10.1016/j.molcel.2011.08.018 21925379 PMC3199020

[B68] Wang L. Wen Z. Ma H. Wu L. Chen H. Zhu Y. (2021). ‘Long non-coding RNAs ENST00000429730.1 and MSTRG.93125.4 are associated with metabolic activity in tuberculosis lesions of sputum-negative tuberculosis patients’. *Aging* 13 8228–8247. 10.18632/aging.202634 33686954 PMC8034958

[B69] Wang L. Xie B. Zhang P. Ge Y. Wang Y. Zhang D. (2019). ‘LOC152742 as a biomarker in the diagnosis of pulmonary tuberculosis infection’. *J. Cell. Biochem.* 120 8949–8955. 10.1002/jcb.27452 30790332

[B70] Wang M. Mao C. Ouyang L. Liu Y. Lai W. Liu N. (2019). ‘Long noncoding RNA LINC00336 inhibits ferroptosis in lung cancer by functioning as a competing endogenous RNA’. *Cell Death Differ.* 26 2329–2343. 10.1038/s41418-019-0304-y 30787392 PMC6889193

[B71] Wang W. Hu W. Wang Y. An Y. Song L. Shang P. (2020). ‘Long non-coding RNA UCA1 promotes malignant phenotypes of renal cancer cells by modulating the miR-182-5p/DLL4 axis as a ceRNA’. *Mol. Cancer* 19:18. 10.1186/s12943-020-1132-x 31996265 PMC6988374

[B72] Wang Y. Zhong H. Xie X. Chen C. Y. Huang D. Shen L. (2015). ‘Long noncoding RNA derived from CD244 signaling epigenetically controls CD8+ T-cell immune responses in tuberculosis infection’. *Proc. Natl. Acad. Sci. U.S.A* 112 E3883–E3892. 10.1073/pnas.1501662112 26150504 PMC4517270

[B73] Washietl S. Kellis M. Garber M. (2014). ‘Evolutionary dynamics and tissue specificity of human long noncoding RNAs in six mammals’. *Genome Res.* 24 616–628. 10.1101/gr.165035.113 24429298 PMC3975061

[B74] Wei L. Liu K. Jia Q. Zhang H. Bie Q. Zhang B. (2021). ‘The roles of host noncoding RNAs in mycobacterium tuberculosis infection’. *Front. Immunol.* 12:664787. 10.3389/fimmu.2021.664787 34093557 PMC8170620

[B75] Wu Q. Zhong H. Bai H. Liu T. Song J. Wen Y. (2020). ‘Clinical relevance of the lnc-HNF1B-3:1 genetic polymorphisms in Western Chinese tuberculosis patients’. *J. Clin. Lab. Anal.* 34:e23076. 10.1002/jcla.23076 31692082 PMC7083404

[B76] Xu Y. Yu J. Ma C. Gong Z. Wu X. Deng G. (2021). ‘Impact of knockdown LincRNA-Cox2 on apoptosis of macrophage infected with *Bacillus* calmette-guérin’. *Mol. Immunol.* 130 85–95. 10.1016/j.molimm.2020.11.008 33250268

[B77] Yang M. Li J. Deng S. Fan H. Peng Y. Ye G. (2022). Competitive endogenous RNA network activates host immune response in SARS-CoV- 2-, panH1N1 (A/California/07/2009)-, and H7N9 (A/Shanghai/1/2013)-infected cells. *Cells* 11:487. 10.3390/cells11030487 35159296 PMC8834034

[B78] Yang X. Yang J. Wang J. Wen Q. Wang H. He J. (2016). ‘Microarray analysis of long noncoding RNA and mRNA expression profiles in human macrophages infected with mycobacterium tuberculosis’. *Sci. Rep.* 6:38963. 10.1038/srep38963 27966580 PMC5155227

[B79] Yao R.-W. Wang Y. Chen L.-L. (2019). ‘Cellular functions of long noncoding RNAs’. *Nat. Cell Biol.* 21 542–551. 10.1038/s41556-019-0311-8 31048766

[B80] Ye Y. Xu Y. Lai Y. He W. Li Y. Wang R. (2018). ‘Long non-coding RNA cox-2 prevents immune evasion and metastasis of hepatocellular carcinoma by altering M1/M2 macrophage polarization’. *J. Cell. Biochem.* 119 2951–2963. 10.1002/jcb.26509 29131381

[B81] Yi Z. Li J. Gao K. Fu Y. (2014). ‘Identifcation of differentially expressed long non-coding RNAs in CD4+ T cells response to latent tuberculosis infection’. *J. Infect.* 69 558–568. 10.1016/j.jinf.2014.06.016 24975173 PMC7112653

[B82] Zeni P. F. Mraz M. (2021). ‘LncRNAs in adaptive immunity: role in physiological and pathological conditions’. *RNA Biol.* 18 619–632. 10.1080/15476286.2020.1838783 33094664 PMC8078528

[B83] Zhang X. Guo J. Fan S. Li Y. Wei L. Yang X. (2013). ‘Screening and identification of six serum microRNAs as novel potential combination biomarkers for pulmonary tuberc(walzl et al., 2018)ulosis diagnosis’. *PLoS One* 8:e81076. 10.1371/journal.pone.0081076 24349033 PMC3857778

[B84] Zhang X. Liang Z. Zhang Y. Dai K. Zhu M. Wang J. (2020). ‘Comprehensive analysis of long non-coding RNAs expression pattern in the pathogenesis of pulmonary tuberculosis’. *Genomics* 112 1970–1977. 10.1016/j.ygeno.2019.11.009 31756428

[B85] Zhao Z. Zhang M. Ying J. Hu X. Zhang J. Zhou Y. (2017). ‘Significance of genetic polymorphisms in long non-coding RNA AC079767.4 in tuberculosis susceptibility and clinical phenotype in Western Chinese Han population’. *Sci. Rep.* 7:965. 10.1038/s41598-017-01163-y 28424495 PMC5430418

[B86] Zhu S. Wang J.-Z. Chen D. He Y.-T. Meng N. Chen M. (2020). ‘An oncopeptide regulates m6A recognition by the m6A reader IGF2BP1 and tumorigenesis’. *Nat. Commun.* 11:1685. 10.1038/s41467-020-15403-9 32245947 PMC7125119

